# Hypogravity modeling of upper extremities: an investigation of manual handling in the workplace

**DOI:** 10.3389/fphys.2023.1198162

**Published:** 2023-10-02

**Authors:** Tatiana Maillard

**Affiliations:** Space Innovation, Doctoral Program in Civil and Environmental Engineering, Swiss Federal Institute of Technology in Lausanne, Lausanne, Switzerland

**Keywords:** reduced gravity, markerless motion capture, motion analysis, joint angles, workplace design

## Abstract

Experiments on the lower limbs are the only approaches being used to study how hypogravity (HG) (0 < g < 1, e.g., Moon: 1/6 g, Mars: 3/8 g) affects human movement. The goal of this study was to expand this field experimentally by investigating the effect of HG on the upper extremities during one-handed manual handling tasks in a sitting posture: static weight holding with an outstretched arm, and slow repetitive weight lifting and lowering motions. The hypothesis was that while completing static and dynamic tasks with elements of repetition in HG, the upper body’s tilt (angle regarding the vertical axis) would change differently from Earth’s gravity. Specifically, upper arm and spine angles, joint torques, and forces were investigated. Twenty-four healthy participants aged 33.6 ± 8.2 years were involved in the trial. Joint angles were examined using vision-based 3D motion analysis. According to this investigation, there is a correlation between a body tilting backward and a gravity level reduction (*p* < 0.01). Thus, HG causes postural deviation, and this shows that workplace design must be adapted according to the level of gravity to promote comfortable and balanced body alignment, minimizing stress on muscles and joints. To lower the risk of musculoskeletal disorders (MSDs), enhance overall performance, and increase job satisfaction, proper support systems and restrictions for sitting positions should be taken into account, concerning different levels of gravity.

## 1 Introduction

Creating a new workplace, seat, and restraining system for astronauts is among the most crucial steps in the overall design process, especially when space missions and operations are still under development and require integration of the developed technical solutions into this design.

Following biomedical research, when designing a workplace, it is necessary to consider the conditions of hypogravity (HG) (specifically on the Moon (1/6 g) and Mars (3/8 g)); this is because ignoring the effects of gravity can lead to health problems and physiological changes (Reynolds, 2019). The following general consequences of HG for the human body in the workplace can be considered from the experience of working in microgravity (MG):

• Musculoskeletal disorders (MSDs). Loss of muscle mass and bone mineral density, as well as curvature of the spine due to a reduced gravity compared to that of Earth, can lead to MSDs such as back pain, spinal cord compression, and osteoporosis (Orwoll et al.; Bloomberg et al., 2016; White et al., 2016; Lang et al., 2017).
• Balance and coordination problems. Changes in the sense of balance and coordination can lead to an increased risk of making mistakes operating in the workplace and other accidents (White et al., 2016). Less is known about how HG affects sensory and motor functions, compared to the effects of prolonged exposure to MG, as suggested by White et al. (2016).
• Cardiovascular issues. Slower blood circulation can lead to cardiovascular problems, including decreased blood volume and reduced heart muscle strength (Barratt and Pool, 2008; Hamilton, 2008; Clément, 2011). According to Richter et al. (2017), physiological measures of cardiovascular effectiveness can be enhanced because cardiac output increases with decreasing gravity levels. The effect on cardiac activity under the influence of lunar gravity has not been investigated yet.
• Vision impairment. Changes in intracranial pressure can result in a modification to visual clarity and optic nerve head edema (Seedhouse, 2015; Özelbaykal et al., 2022).

At present, little is known about upper limb motion under HG as most of the experiments are limited to those performed on the lower limbs (Richter et al., 2017; Hewes and Spady, 1964; Rajulu and Klute, 1992; Newman and Alexander, 1993; Sylos-Labini et al., 2014; Kang et al., 2019; Weber et al., 2019). To the author’s knowledge, studies of sitting positions when performing manual handling with operational weights under HG have recently been reported only once in a publication (Volkova et al., 2022) and thesis (Volkova, 2022). The lack of data and objective measures of upper limb and trunk movements during task performance generates difficulties in evaluating movements and proposing the measures to mitigate the effects. Compared to Earth, there is little evidence that short- or long-term missions have an impact on functional changes in upper limb movement in HG. In this regard, this study’s focus was on the upper extremities’ biomechanics, particularly in the sitting posture under HG circumstances.

The principles of ergonomics of sitting must be respected under HG conditions as they are on Earth. It is important to investigate the most critical manual tasks, such as hazardous tasks that can stress the body and lead to MSD. There should be less stress from the load on the muscles and joints while performing any manual tasks in a seated position. The choice of the layout of the workplace should help reduce these loads. Tasks such as lifting, lowering, carrying, and transferring weights can be hazardous, if they require uncomfortable postures, and involve repetitive, sustained, or high force. In this paper, the term “manual handling” was used instead of hazardous manual handling in accordance with New Zealand and European Union (Directive, 1990; Occupational Safety and Health Service, 2001).

Biomechanics analysis tools have evolved significantly due to the ability to combine complex models of the human body, computer vision, and machine learning algorithms (Colyer et al., 2018). In this context, markerless 3D upper limb kinematics with motion capture is not commonly used for motion studies due to the perceived technical difficulties in creating a representative model and in identifying the required task and motion of interest (Colyer et al., 2018). However, markerless motion capture offers numerous advantages because it is simple to use and non-intrusive (Mündermann et al., 2006; Kanko et al., 2021). Moreover, according to Nakano et al. (2020), marker-based and markerless motion capture methods have relatively nearly the same small mean absolute error (MAE).

This study’s objective was to identify the differences between the upper limb and trunk movements in relation to the vertical axis in simulated HG and terrestrial gravity when performing tasks in a sitting position. Then, in accordance with the found results, the strategies for eliminating or minimizing the risks, including MSD associated with manual handling, were identified.

The following dangerous manual tasks were identified in this work: sustained static and repetitive lifting, and lowering holding of operational weights with one hand. All participants conducted these tasks until fatigue failure occurs. Then, a 3D kinematic analysis of joint angles and joint torques, and forces is assessed to find the difference in the motions under Earth and lunar gravity to define the difference in the motions, particularly the trunk and upper arm tilt concerning the vertical axis. Determining and comparing participants’ body sitting positions (specifically body tilt) while performing tasks under different gravity conditions allows not only to see the effect of gravity levels on the body movements but can also help engineers make decisions on the design of seating workplaces and tasks, as well as related movements performed under lunar gravity conditions. Such movements should not be constrained to reduce stress on the body. By avoiding parasitic stress on the body, considering the new body position at the workplace under HG, health problems can be prevented and a safe and healthy working environment for astronauts in the lunar environment can be created.

## 2 Materials and methods

### 2.1 Participants and protocol environment

Twenty-four individuals took part in the study (12 male and 12 female participants). The number of participants is based on a statistical calculation of the size effect, as shown in Section 1.1, Supplementary Material. This cohort is also based on the 12-astronauts base used in all long-distance flight programs. Table 1 shows descriptive anthropometric data on the participants. To determine whether participants did not already have health issues, all participants were required to complete a survey (Warburton et al., 2019), Supplementary Figure S1. There were seven simple health-related questions in this survey, and there were only two viable responses: “Yes” or “No.” Only those who responded “No” to each of the questions were invited to participate. In a 1 g environment and then underwater with simulated HG, similar experiments with static and dynamic/kinematic tasks were conducted. Buoyant force counteracts the gravitational force; hence, underwater conditions were utilized to simulate lunar gravity (g = 1.626 m/s^2^).

**TABLE 1:** Descriptive anthropometric data on the participants in the experiment. SD, standard deviation. Upper arm (m)—the distance between the point of the upper arm and forearm; forearm (m)—the distance between the point of the forearm and wrist.

Study variable	Total (N = 24)	Min/Max	Male individual (N = 12)	Female individual (N = 12)	*p*-value
	Mean (SD)		Mean (SD)	Mean (SD)	
Age (yr)	33.59 (8.16)	25/55	34 (9.62)	33.07 (6.11)	0.742
Height (m)	1.75 (0.11)	1.54/1.95	1.83 (0.07)	1.66 (0.06)	<0.001
Body mass (kg)	71.22 (17.01)	43.8/114.10	82.92 (13.02)	56.19 (5.95)	<0.001
Upper arm (m)	0.34 (0.04)	0.25/0.40	0.35 (0.33)	0.32 (0.03)	0.007
Forearm (m)	0.28 (0.03)	0.20/0.33	0.30 (0.02)	0.25 (0.02)	<0.001
Torso volume (dm³)	37.00 (11.00)	28.00/61.00	44.71 (6.93)	27.00 (4.42)	<0.001
Upper arm volume (dm³)	2.00 (0.80)	0.8/3.8	2.70 (0.67)	1.48 (0.38)	<0.001
Forearm volume (dm³)	1.00 (0.30)	0.4/2.00	1.37 (0.20)	0.72 (0.16)	<0.001

**FIGURE 1:**
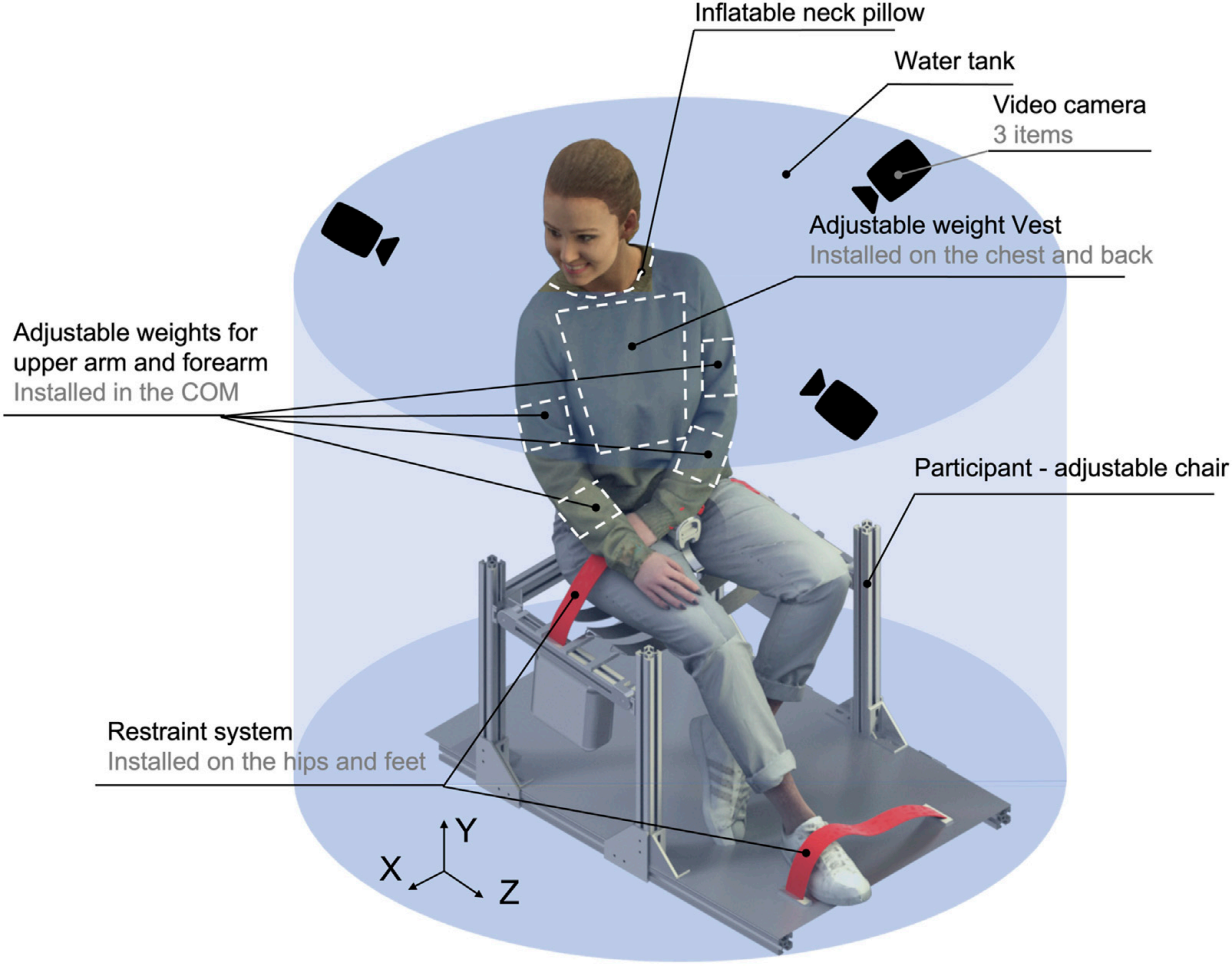
Experimental setup. COM, center of mass. The mannequin (female) image is from the open-source model https:/humano3D.com.

During the studies in the part of the diving center adapted to the water tank (Swissub, Vaud, Switzerland), the participants were seated with their heads above the water, as shown in Figure 1. This water tank has an 8.3-ton capacity. The water’s temperature remained unchanged at 29°. Forearm, upper arm, and torso adjustable weights were chosen to produce the required level of buoyancy, which is equivalent to gravity on the Moon. All participants signed a written informed consent form before the experiments. This form, as well as each step of this study, was approved by the Ethics Committee of the Swiss Federal Institute of Technology in Lausanne (HREC 024-2021/09.03.2021 amendment to initial protocol HREC 001-2020/20.12.2019).

### 2.2 Experimental setup

The experimental setup included hardware and software parts. Hardware was needed to create a seated workplace and to take some baseline measurements of the participants (height, mass, and upper limb volume). A model of distributed adjustable body weights was also developed to create the required level of buoyancy for lunar gravity simulation. The software part was needed to continue extracting the main input information on the participants, such as the volume of body parts (head and torso parts). The software part was also used to calibrate the camera and process data after video recording and extract parameters related to the movement of participants.

#### 2.2.1 Hardware part

**FIGURE 2:**
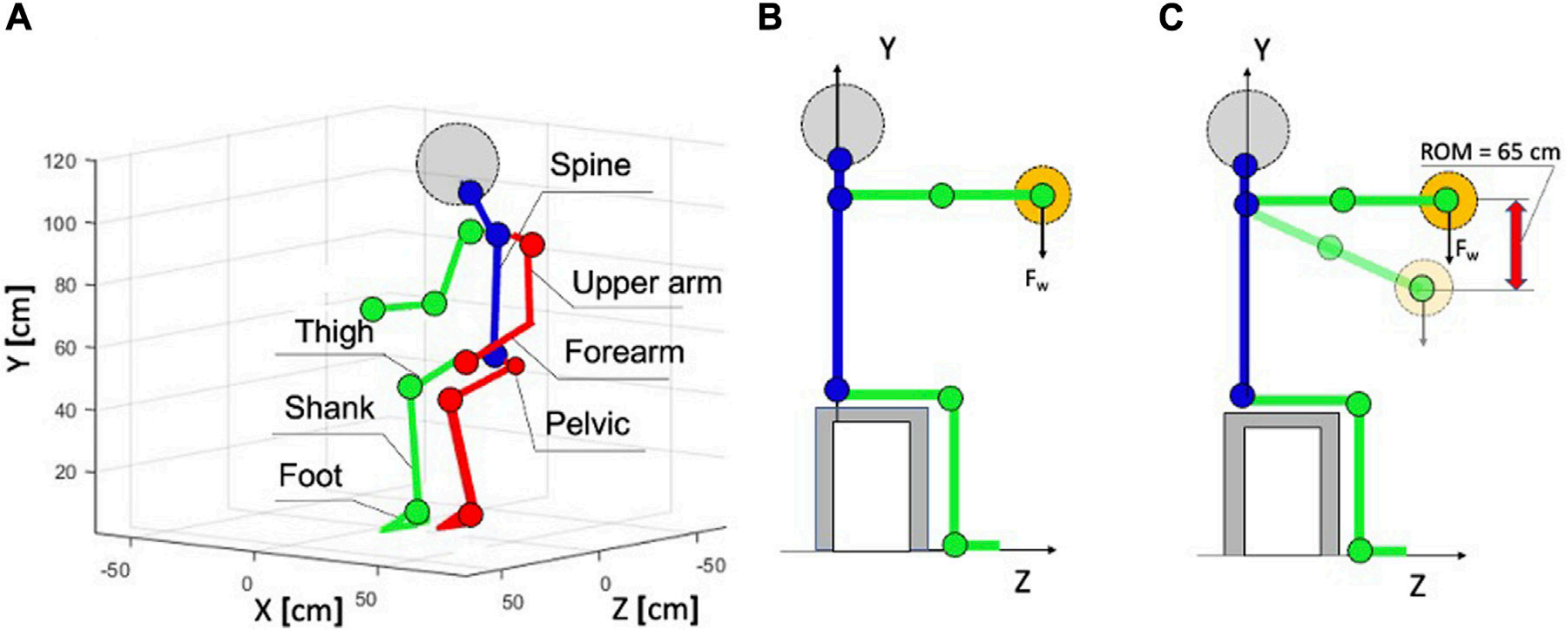
**(A)** Sitting skeleton coordinate system with the definition of body parts. **(B)** Static weight holding task with an outstretched arm. **(C)** Dynamic/kinematic tasks with elements of repetition (weight lifting and weight lowering). ROM, range of motion.

Participants’ height was determined using a stadiometer (NutriActivia, Minnesota, United States). Their mass was found with a bioelectrical impedance analysis scale (Nokia Health, Body +, China). The custom-made water displacement method was used to measure the volume of the hand, forearm, and upper arm volume measurement, as shown in Supplementary Methods 1.2. The definition of body parts is presented in the Figure 2A. This method was used in connection with the possibility of increasing the speed of measurements since the software assessment of the volume of the upper limbs required laborious refinement of the digital mesh.

In the context of this research, a static weight-holding task with an outstretched arm and a slow (max 47 cm/s) dynamic task with elements of repetition, such as lifting and lowering weights with a range of motion (ROM) equal to 3 s ( ∼ 65 cm), were considered for video recording sessions, as shown in Figure 2B. The masses of operational weights with which the participants worked at the workplace when performing tasks were 1 kg and 3 kg. The chosen tasks are of decisive importance for the astronauts. Static tasks with loads can be found in the repair and maintenance tasks of the electronics and construction industry, while dynamic tasks with repetitive elements are found in assembly lines, during the assembly and manipulation of small- and medium-sized components. These tasks are accompanied by muscle work that causes fatigue and decreased performance even at very low operational weights. The selected operational weight range (1–3 kg) accurately reflects the typical weight of items that working astronauts (male and female individuals) may regularly work with. They are suitable for a variety of everyday manual tasks, making them relevant to a wide range of workplaces. By studying these tasks, potential injury risks can be identified, and preventive measures can be developed, including training, equipment adaptation, and task interleaving.

An experimental setup with a participant-adjustable chair was built from the stainless steel profiles (Item, Germany). All participants wore leg and hip straps attached to this chair to avoid the motions of the lower part of the body. The participants’ legs were attached to the footrest as per anthropometry, considering the length of the legs from knee to foot. Three video cameras were used for the video recording of vision-based processes (GoPro 8, Woodman Labs, Inc., San Mateo, California, United States). GoPro specifications comprised 1,920 by 1,080 at 30-Hz (4 k condition) resolution.

For use in underwater investigations, a numerical simplified model of adjustable weights was created, which was designed for different parts of the body (upper part) of the participants. This model is described in detail in the main author’s study (Volkova et al., 2022) and the same author’s dissertation (Volkova, 2022). Adjustable weights for the upper arm and forearm (Strong shop. ch, Switzerland) were comfortable wearable straps with many velcro-based pockets. The buckle was used to adjust the diameter to match the participants’ upper arm or forearm diameter. Each pocket of the wearable strap was filled with prefabricated, fully sealed weights filled with high-density iron sand by the required level of buoyancy, measured mass, and the volume of the upper arm and forearm of the participants. These adjustable weights were installed in the center of mass (CoM) of the body parts. A weighted vest (THORN + Fit Schweiz, Basel, Switzerland) was used for the torso. It was a vest with a similar wearable strap system, multiple pockets, and velcro-based closure. Pockets were distributed on the chest and back of the participants. The velcro straps were used to adjust the diameter of the vest to the diameter of the participants’ chests. Weight pockets were also tailored to the parameters of each participant and distributed equally on the chest and back as close as possible to the CoM of the participants’ torso.

#### 2.2.2 Software part

**FIGURE 3:**
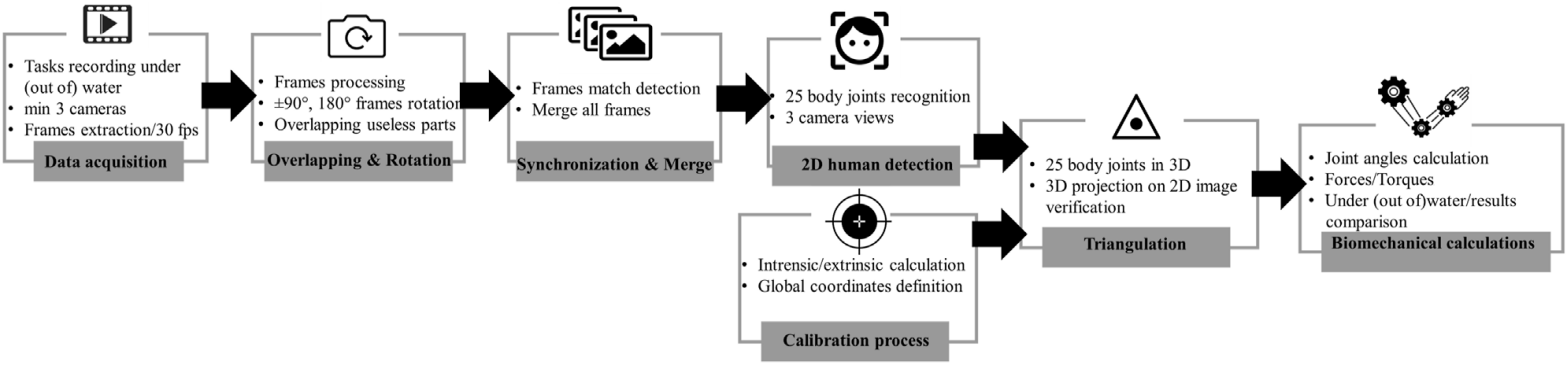
Data processing scheme for the markerless motion capture method and biomechanical computation. Modified from Volkova (2022).

This part required the development of an individual approach and a combination of software and steps to be able to extract the target output. The data processing steps of this part are shown in Figure 3. Markerless motion capture using a deep learning technique was applied with the support of OpenPose (version 1.4.0) software (Cao et al., 2017; Simon et al., 2017). It was installed from the “OpenCV docs website” (OpenCV, 2022). To be able to run this software, a graphics processing unit (GEFORCE GTX 2080; Nvidia Corp, Santa Clara, CA, United States) was used for data processing. OpenPose has a different pose output format for JSON mapping. For this study, Body_25 was selected, which represents 25 joints of the full body skeleton of participants. Such an output was saved for each frame of the experimental video and stored in the JSON format.

The cameras were calibrated with a calibration tool script created by the computer vision laboratory at the Swiss Federal Institute of Technology in Lausanne, “Github CVLab” (CVLab, 2022). This calibration tool allowed manual annotation of control points to create a common coordinate system for all participants and to find the main elements of the workplace on video images.

Then, the extraction and implementation of intrinsic and extrinsic data were performed for 3D posture reconstruction. Errors in camera calibration, such as global registration and bundle adjustment, were computed. For assessing the quality of joint recognition, the algorithm of each joint was compared, and then, the algorithm of each joint was calculated. The experimental room light was used to synchronize frames on all cameras. The cameras were started with the light off, and then, the light was turned on by the experimenter before the start of the experiment and at the end of each task performed by the participant.

The useable frames for each camera were manually determined using FFMPEG software (Newmarch, 2017). The definition of the synchronization frame made it possible to find a total equal number of frames for each video camera. The manual selection of frames was based on the criteria that the frames showed participants completing tasks from the start to the end of the task. The first frame represents picking up the weight and being ready to begin the activity, while the last frame represents the participant’s exhaustion and inability to continue with the experimental technique.

For the created adjustable weight model, it was necessary to estimate the volume of the torso of participants. Agisoft Metashape 1.7.2 software (Agisoft Ltd., St. Petersburg, Russia) was used to calculate the volume of the torso because it was quick and non-invasive for the experiment’s participants (Jebur et al., 2018). For each participant, 1,000 photographs were collected using a high resolution (12 megapixels), focal length (4.25 mm), and f/1.8 cameras. Calculations were performed using a digital surface model resolution of 10 cm/pixel with good or medium cloud quality. First, a photogrammetry-generated body mesh of participants, called a modified mesh, was imported into Blender 2.8 (Blender Foundation, Amsterdam, Netherlands). A target mesh was then added for each body segment, and the Boolean modifier allowed operations on complex 3D meshes to be performed. These operations include options for intersecting differences and unions. The difference operation allows the subtraction of the target mesh from the changed mesh (Freixas et al., 2006) of a specific body segment. Participants’ full body volumes were scaled based on their height. The asymmetric mesh was occasionally modified via Blender proportional editing^1^ function (Guevarra and Guevarra, 2020). According to Volkova et al. (2021), this method gives a relatively small difference in the 3D body model estimate for the total body volume compared to a water-based body volume measurement.

### 2.3 Statistical methods

Statistical analysis software R (4.1.3) and Excel were both used to study the distribution of data (R Foundation, New Zealand). The G*Power software version was used to calculate effect sizes (3.1.9.7). In this study, task duration and angle-related position data were considered the basis for analysis. The joint angle data were examined as means and lowest and maximum indicators at the start, halfway, and finish of the task, respectively. The end time of the task was equal to the endurance time, the point at which muscle fatigue sets in following the loss of the participant’s ability to produce force. The values for the whole endurance time for male and female individuals per task are presented in Table 2. These values determine the period that the participants worked until fatigue failure. After several sorting of the data, it was decided to combine static and dynamic/kinematic tasks into one dataset due to the weak dependence of the task and body inclination with decreasing gravity, rather than the dependence of the human gender on body inclination with decreasing gravity. The data were analyzed with the Shapiro–Wilk test. According to the central limit theorem (Kwak and Kim, 2017), the data were expected to be normally distributed, even though the experiment focuses on human factors. In addition, this test was used to test for normality. Since the number of observations is not so large, a measurement error cannot arise due to the sensitivity of the test to small deviations. A normal distribution of the data is shown if the *p*-value >0.05.

**TABLE 2:** Endurance time (ET) for static (S) and dynamic tasks with repetitive element (D) tasks under 1 g and 1/6 g for male/female individuals. Adapted from Volkova (2022). Creative Commons license.

Task-gravity level	Mean ET (min) load (1 kg)	Mean ET (min) load (3 kg)
22 participants		
S—1 g	1.67/0.95	0.85/0.34
S—1/6 g	7.73/6.21	2.65/0.65
25 participants		
D—1 g	1.30/0.80	0.79/0.35
D—1/6 g	14.93/9.34	2.16/0.82

The threshold for statistical significance was = .05. A *post hoc* power analysis indicates that with all of the participants divided into two groups, a power of 0.80 for average-sized group effects can be found. Section 1.1 of the Supplementary Material contains the power analysis discussion.

According to statistical information (Plagenhoef et al., 1983), participant body parts’ masses and lengths were measured. According to the same author’s statistical data, the CoM of the body segments was estimated, as shown in Supplementary Figure S2 and Supplementary Table S1.

### 2.4 Angle assessment between the spine/upper arm and vertical axis

**FIGURE 4:**
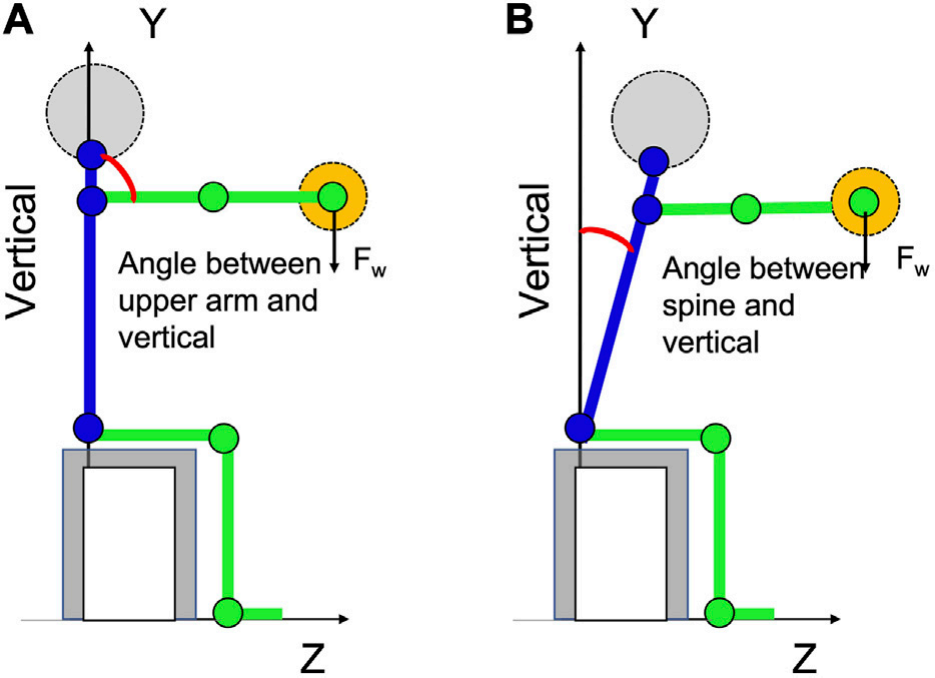
**(A)** Measured angle between the upper arm and vertical axis. **(B)**Measured angle between the spine and vertical axis.

The joint angles for 3D postural assessment were evaluated using a custom-made MATLAB script. This script required the following input: the number of the participant, the range of the frames necessary for data processing, the gravity level, and the operational load level in kg. The MATLAB script is based on triangulated 2D skeletons recognized by OpenPose results and camera calibration output. At each stage of task execution, the software application recognized all rigid links and joints of the participants. In this study, the angle between the spine, upper arm, and vertical axis Y was considered, as shown in Figure 4. Other measured angles are described in the work of the main author (Volkova, 2022). They are not described here because these angles did not give significant results for the development of further hypotheses related to workplace design under HG. The study of how the human body, for example, through the tilt of the body, responds to different weights, at different levels of gravity, expands the understanding of the effect of gravity on task performance.

### 2.5 Joint forces and torques assessment

The Lagrange equation (recursive dynamics techniques) was used as the foundation for the dynamic motion equation. It was chosen since the increased numerical performance stability is provided by recursive dynamics (Hollerbach, 1980). The Lagrangian approach, akin to a robotic arm’s dynamics, exhibits robustness in dynamic representation. Although precision is context-dependent, qualitative behavior remains reliable. It was supposed that the only forces acting on participants’ rigid links were joint torques, operating weights, and forces of gravitational acceleration. The range of possible hand movements was narrowed down to vertical axis raising and lowering (a dynamic/kinematic task with the elements of repetitive motions) of one hand with two degrees of freedom. The CoM value of the upper arm and forearm was determined by the model described previously (Plagenhoef et al., 1983). For the length of the shoulder and forearm, the real values ​​of the participants in the experiment were used, as shown in Table 1. For such movements, the following Lagrange equations (Hollerbach, 1980) were used to calculate the actuation torques of the joints:

$$\begin{array}{ll} \tau_{1} & =\left(I_{1}+I_{2}+m_{1}l_{1}^{2}+m_{2}\left(L_{1}^{2}+l_{2}^{2}+2L_{1}l_{2}\cos\theta_{2}\right)\right)\ddot{\theta_{1}}+\left(I_{2}+m_{2}l_{2}^{2}+m_{2}L_{1}l_{2}\cos\theta_{2}\right)\ddot{\theta_{2}}-2m_{2}L_{1}l_{2}{\dot{\theta }}_{1}{\dot{\theta }}_{2}\sin\theta_{2}\\ & -m_{2}L_{1}l_{2}{\dot{\theta }}_{2}^{2}\sin\theta_{2}+m_{2}gl_{2}\cos\left(\theta_{1}+\theta_{2}\right)+m_{1}gl_{1}\cos\theta_{1}+m_{2}gL_{1}\cos\theta_{1}+fL_{2}\cos\left(\theta_{1}+\theta_{2}\right)+fL_{1}\cos\theta_{1} \end{array}$$

$$\tau_{2}=\left(I_{2}+m_{2}l_{2}^{2}\right){\ddot{\theta }}_{2}+\left(I_{2}+m_{2}l_{2}^{2}+m_{2}L_{1}l_{2}\cos\theta_{2}\right){\ddot{\theta }}_{1}+m_{2}L_{1}l_{2}{\dot{\theta }}_{1}^{2}\sin\theta_{2}+m_{2}gl_{2}\cos\left(\theta_{1}+\theta_{2}\right)+fL_{2}\cos\left(\theta_{1}+\theta_{2}\right)$$

where $\ddot{\theta_{1}},\ddot{\theta_{2},}$ and ${\dot{\theta }}_{1},{\dot{\theta }}_{2}$ are gradients of torque;

***L***_***1***_ and ***L***_***2***_ are the lengths of the upper arm and forearm, respectively; ***l***_***1***_ and ***l***_***2***_, are the lengths between the upper arm joint and the CoM of the upper arm and the joint of the forearm and the CoM of the forearm, respectively; ***I***_***1***_ and ***I***_***2***_ are the moments of inertia of the upper arm and forearm, respectively.

## 3 Results

### 3.1 Pose estimation

According to the Shapiro–Wilk test, all task data are regularly distributed, as shown in Supplementary Table S2. The accuracy of OpenPose was previously assessed and discussed in the thesis of the main author “Biomechanics at the Workplace under Hypogravity” (Volkova, 2022). In this article, the author provides the main results confirming the accuracy of OpenPose in a aquatic environment. The percentage of correct key points (PCK) was 100 for 1 g and 77 for 1/6 g per four selected frames. The unseen joints were not included in the PCK results. For 1 g and 1/6 g, the mean absolute error (MAE) shoulder was 4.3 mm and 9.1 mm, respectively. In addition, MAEy’s shoulder measured 3.6 mm for 1 g and 11 mm for 1/6 g (Volkova et al., 2021). For the evaluation of the accuracy of the global skeleton pose estimate, mean per joint position errors (MPJPEs) of 20.4 for 1 g and 25.5 for 1/6 g were found (Volkova et al., 2021). For MPJPE, global coordinates, as well as coordinates between the shoulder and elbow, were used to estimate the joint error. The recognition accuracy of the markerless motion capture method can depend on many factors. The optical properties of water, such as refraction and reflection, can distort the results. In addition, water conditions can affect light penetration and change the visibility of the body and scene, resulting in less accurate motion capture. Furthermore, the accuracy may depend on the pattern of the upper garments worn by people. Dark monotonous garments impair recognition. Another possible source of errors can be related to data processing, as well as incorrect time synchronization of cameras.

### 3.2 Angles between the spine/upper arm and vertical axis

The analyses are performed to examine the effects of two different environments (1 g and 1/6 g) on the observed indicators, such as the upper arm and spine angles. In the context of Lagrange’s dynamics, these values are vital for both observing the system (measures and models) and controlling it through muscular actions. They serve as essential elements determining the system’s observability and commandability. Over the course of the participant’s completion of the task, the values of the indicators were considered at the beginning of the exercise, the first 5 s, the average moment which corresponds to half the full endurance time of a particular participant, and the moment when the participant is completely tired (the last 5 seconds before the end of the exercise).

**FIGURE 5:**
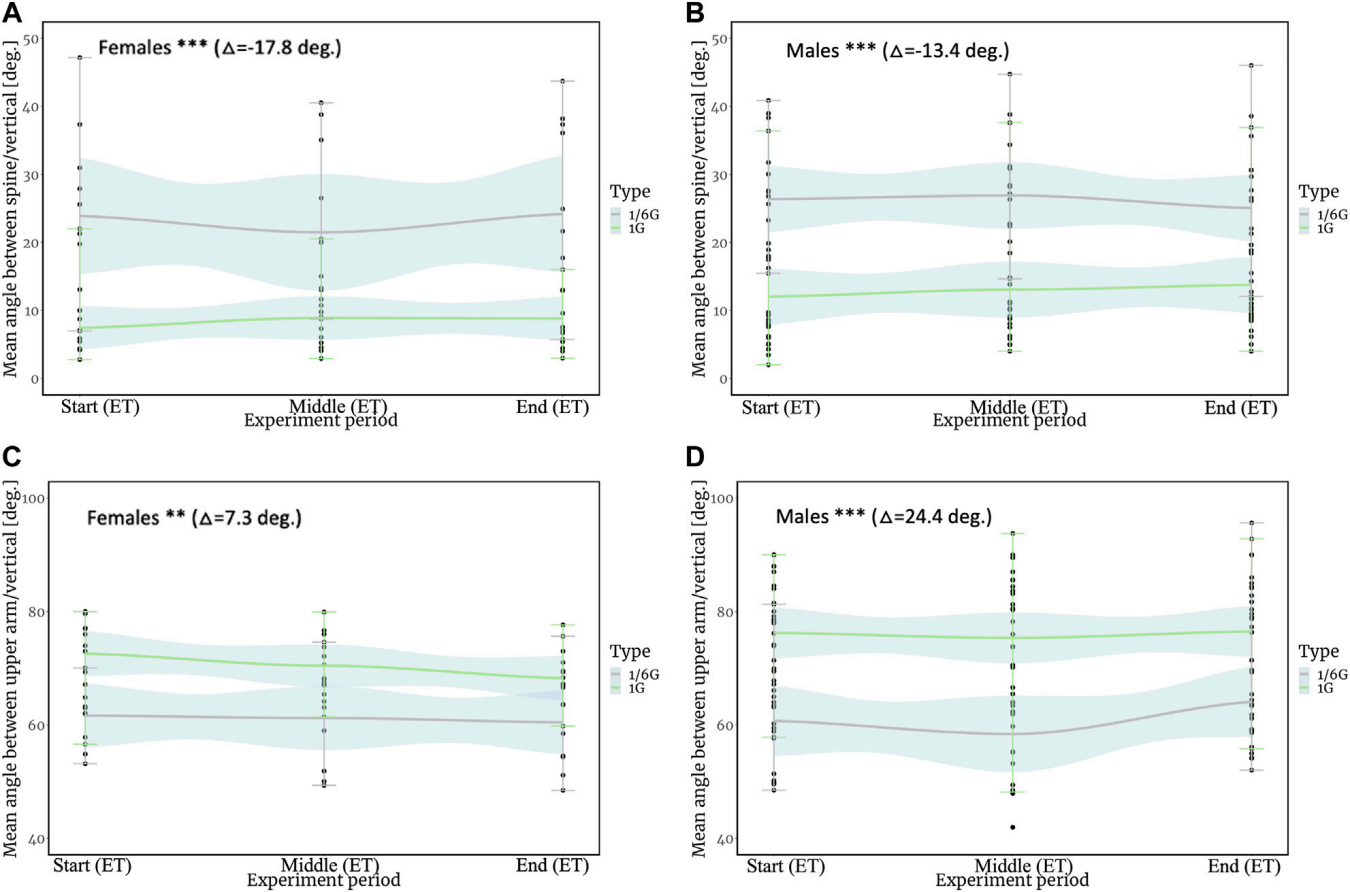
**(A)** Results for the mean angle between the spine and vertical axis for female individuals (*Y*-axis). **(B)** Results for the mean angle between the spine and vertical axis for male individuals (*Y*-axis). **(C)** Results for the mean angle between the upper arm and vertical axis for female individuals (*Y*-axis). **(D)** Results for the mean angle between the upper arm and vertical axis for male individuals (*Y*-axis). Gravity changes from 1⁄6 g to 1 g. Delta (△**)** corresponds to the change in angle as gravity increases from 1/6g to 1 g. ET, endurance time. ***p < 0.1; **p < 0.05; *p < 0.01. Modified and adapted from Volkova (2022).

Figures 5A,B depict the angles between the spine and the vertical axis for male and female individuals separately (holding weight 1 kg and 3 kg). The results were presented for male and female individuals separately for combined datasets for static and dynamic/kinematic tasks as a consequence of the fact that the primary statistical calculations showed that the gender of the participants in the experiments affects the change in the result, while the type of task almost does not affect the results. Details of the statistical calculations of regressions for angle variables are presented in Supplementary Table S3. The results for 1 g and 1/6 g are shown on the same plot for comparative reasons. The endurance time values, corresponding to the specific environment, task, and gender are presented in Table 2. When executing the same tasks, the female and male individuals' angles are larger under 1/6 g than those under the Earth’s gravity, according to these figures. Under 1/6 g, this angle on average is 17.8° larger for female individuals and 13.4° larger for male individuals as compared to Earth’s gravity.

Figures 5 C, D illustrate the angle between the upper arm and the vertical axis. The results were presented for male and female individuals separately for the same reason as the results for the angle between the spine and vertical axis. Thus, these are combined datasets for static and dynamic/kinematic tasks. For dynamic/kinematic tasks, the average angle of the upper position of the arm was considered. The found data indicate that the angle between the upper arm and the vertical axis is slightly smaller under the simulated lunar gravity. Under 1/6 g, this angle is on average 7.3° smaller for female individuals and 24.4° smaller for male individuals as compared to Earth’s gravity.

### 3.3 Joint forces and torques

The magnitude of torques and forces applied to the joints shows the load on the muscles under experimental conditions at HG and Earth’s gravity. Due to the method’s intricacy and attention to upper extremity limb studies, the calculation was only able to calculate torques in the elbow and shoulder joints for problems involving static postures and dynamic/kinematic motions.

The results for forces and torques are presented in Table 3. It shows the values for elbow and shoulder joints for 24 participants at two distinct gravity levels, 1 g and 1/6 g. The results (mean and standard deviation) are shown separately for male and female individuals for static and dynamic/kinematic tasks.

**TABLE 3:** Forces (N) and torques (NM) of the shoulder and elbow joints for static (S) operational weight holding with outstretch arm holding (1kg, 3 kg) and slow dynamic/kinematic tasks with repetitive elements( D) for male (M)/female (F) individuals. All values are presented as mean **±** **standard deviation (SD). Adapted from Volkova (2022). Creative Commons license.**

Joint—Task—gravity	Force, N (mean ± SD), M/F	Torque, N·m (mean ± SD), M/F
**Shoulder—S—1g**	56.22 ± 11.76/41.98 ± 10.33	18.91 ± 8.18/14.91 ± 5.14
**Shoulder—S—1/6g**	30.18 ± 10.72/20.97 ± 2.76	9.03 ± 6.87/2.34 ± 1.44
**Elbow—S—1g**	29.82 ± 10.28/26.82 ± 11.28	6.53 ± 2.79/5.11 ± 2.52
**Elbow—S—1/6g**	25.9 ± 10.28/10.61 ± 2.93	5.07 ± 3.34/0.97 ± 0.67
**Shoulder—D—1g**	59.57 ± 10.12/40.13 ± 10.01	19.45 ± 7.56/13.15 ± 3.51
**Shoulder—D—1/6g**	23.79 ± 9.56/9.02 ± 6.52	4.8 ± 5.78/2.64 ± 2.54
**Elbow—D—1g**	32.63 ± 7.58/25.41 ± 6.58	6.22 ± 3.57/4.88 ± 2.51
**Elbow—D—1/6g**	19.43 ± 9.52/6.42 ± 5.53	3.48 ± 2.56/1.09 ± 0.25

## 4 Discussion

Because workers get tired more easily when they have an awkward posture at the workplace, which lowers productivity, maintaining a body posture is crucial (Haynes, 2008). The objectives of this study were to identify various participant postures using biomechanical modeling and to suggest enhancements in associated task productivity. To achieve this, the study explored how markerless motion capture can be used to identify changes in participants’ sitting postures at joint angles (between the spine/upper arm and vertical axis) in a simulated HG environment and in Earth’s gravity to compare results. In addition, the analyses in this study resulted in a description of parameters such as forces and torques of the upper extremities.

Comparing the angles between the spine and the vertical axis (*Y*-axis) revealed a peculiar phenomenon. From this analysis, a significant (*p* < 0.01) mean angle variation between the spine and vertical axis under HG was found in comparison with 1 g for male and female individuals. For males and females, the greatest deviation was observed in the aquatic environment. For female individuals, this deviation averaged 17.8° in the absolute value in water in comparison with that of land. For male individuals, the deviation in water averaged 13.4° in comparison with that of land. The minimum and maximum values of the deviation of the angle from the vertical axis in the simulated lunar environment also have a fairly large range in comparison with the results obtained on land. This is probably due to participants of the experiment losing stability when performing the task in water, compared to that on land.

The angles between the upper arm and the vertical axis (the *Y*-axis) decreased in both males and females when performing static and dynamic/kinematic tasks with elements of repetition, while HG participants tended to gently lift their arms during the duration of the tasks, as evidenced by a comparison with 1 g. Male and female individuals raised their arms by 24.4 and 7.3°, respectively, during HG compared to 1 g. The significance of this divergence regarding the increase in gravity was less significant (*p* < 0.05) for female individuals and more significant (*p* < 0.01) for male individuals. This is most likely due to the arm’s weight in the water being significantly lower (even despite the distributed wearable weights on the upper arm and forearm) than the operating load due to the simulated lunar gravity. By comparing the data, the angles between the upper arm/spine and the vertical axis differ significantly depending on the environment. The *p*-value, which reflects how significant the deviation is generally, served to highlight the significance of this discrepancy. It is, therefore, seen that the conditions of reduced gravity to a large extent influenced the change in the position of the body under load in space. This is probably because the lightening of the body of the participants in water affects the position of the body with a slight deviation back and the likely displacement of CoM down the vertical axis. This position appears to help participants stabilize their bodies and reduce rotation while performing the tasks. The results should be verified in real-world scenarios because there may be numerous types of inaccuracies related to forecasting ability (for example, parabolic flight experiments).

Utilizing the markerless method of motion capture and biomechanical modeling, it was feasible to determine the torque and forces in the upper extremity joints, which is a measure of the strain on the muscles, using the anthropometric and kinematic data on the participants. The muscle load under the HG experiment settings and the Earth’s gravity with a different mass of operational weights were compared using data on the magnitude of the torque. The values were found for the shoulder and elbow. Comparing the values of torques and force in simulated lunar gravity and on 1 g it can be seen that the load on the muscles under 1/6 g is reduced. It is, therefore, seen that the conditions of reduced gravity to a large extent influenced the change in the position of the body under the load in space.

The use of markerless motion capture can significantly increase the efficiency of posture and motion analysis, and speed up data collection, especially in long-term tasks, even in underwater environments. This method is substantially less expensive than sensor-based techniques, which can significantly enhance the amount of data gathered and, as a result, the outcomes. In addition, this method can be used to analyze the motion related to the tasks that are still in the design phase. Since there are yet no recommendations and decisions on how astronauts will execute their varied operational tasks, this directly relates to the design of lunar and Martian bases, especially working and living spaces. Last, analyzing joint profiles with markerless techniques might help define precisely how one’s body posture changes while working.

The error calculation showed that motion recognition underwater worked a bit less precisely than under land-based conditions, but the difference was insignificant. Tracking errors were addressed in the underwater case study by manually digitizing the joints’ proper positions on 2D frames. By triangulating the 3D models of the participants, the uncertainty about the depth of the 2D posture assessment was removed. These findings unmistakably indicate that this strategy merits testing in the scientific community, particularly under the circumstances covered by this study. The accurate synchronization of frames from several cameras is crucial for the accuracy of 3D pose estimation utilizing markerless motion capture; thus, it must be verified that there are no errors at these phases. The actual data processing itself might be a potential cause of inaccuracy. It is assumed that it is important to introduce additional steps to predict the sitting position of participants’ spines as the current OpenPose algorithm is limited in this aspect and because in the ergonomics of sitting, the spine is a key element.

## 5 Conclusion and perspectives

This biomechanical modeling significance lies in accentuating sitting posture changes in the workplace under HG in comparison with Earth’s gravity, which can have an impact on worker productivity, discomfort, and fatigue. The postural changes, expressed in joint angle variations, were examined through markerless motion capture.

The angle variation between the spine and vertical axis under simulated HG conditions in comparison with Earth’s gravity was highlighted. Under 1 g and 1/6 g, the effects of body loading on different muscles and joints were different, resulting in a different posture profile and the need for different health and countermeasures approaches for these situations. The assessment of torque and force in upper extremity joints unveiled reduced muscle load under simulated HG in comparison with Earth’s gravity, underscoring gravity’s influence on body positioning and muscle dynamics.

Markerless motion capture helped speed up pose analysis, particularly underwater. In addition, this cost-effective method improves data collection, helping to solve design problems that are still under development. It can also be successfully applied in research on sports, gaming, and on rehabilitation procedures. Nevertheless, accurate camera frame synchronization is decisive for accurate 3D pose estimates. If the current problems associated with the accuracy and reliability of markerless motion capture using computer vision are resolved, this method is likely to have an impact on biomechanics study under HG in future. It is suggested that further collaborative work between computer vision experts and biomechanics should be undertaken to develop such methods.

The results and method presented in this paper may positively impact the development of workplace design guidelines and standards related to posture assessment. Based on the developed biomechanical results, individual training programs for astronauts can be developed, aimed at minimizing the number of static and dynamic tasks with loads in the workplace. Understanding upper extremities biomechanics under HG ensures that astronauts can perform their duties efficiently and safely, preventing MSD under a variety of working conditions in the gravitational environments of the Moon and Mars. The obtained results are recommended to be validated in real scenarios, such as parabolic flights simulating HG environments.

In summary, this study dedicated to posture, gravity, and muscle dynamics plays a crucial role for improving workplace design, training, and overall human wellbeing. The future directions with promising perspectives can be related to the study, where posture is paramount, i.e., pilots, fixed-point work with repetitive movements, and individuals with reduced mobility.

## 6 Limitations

The technical limitation of 3D analysis of upper limb movements lies in the reproduction of large degrees of freedom in the shoulder complex and the elimination of potential motion errors in the movement of the forearm and the upper arm. Another limitation is related to how the skeletal model of the OpenPose does not consider details of the back but rather represents it as a straight line. All results related to the analysis of movement in the underwater environment must be validated using parabolic flight.

## Data Availability

The datasets presented in this study can be found in online repositories. The names of the repository/repositories and accession number(s) can be found in the article/Supplementary Material.

## Footnotes

1 The proportional editing function (Blender software) allows the changing of certain digital model elements (such as mesh elements) with a smooth scaling impact
