# Supplementary material for: Hypogravity modeling of upper extremities: an investigation of manual handling in the workplace
Source: Front Physiol. 2023 Oct 2;14:1198162. doi: 10.3389/fphys.2023.1198162 (PMC10580979; doi:10.3389/fphys.2023.1198162)
Supplement: Supplementary file 1 [file DataSheet1.docx]

Supplementary Material

Hypogravity modeling of upper extremities: an investigation of manual handling in the workplace

Tatiana Maillard*

*** Correspondence:** Corresponding Author: tatiana.maillard@alumni.epfl.ch

# Supplementary Data

Supplementary materials include information related to the participant’s hiring procedure, including participants descriptive statistics.

## Supplementary Material (Power analysis and Size effect)

This experiment was planned for 2 independent groups, each undergoing 2 different conditions. Across two experimental groups, thresholds were established as follows: 0.2 (little effect), 0.5 (moderate effect), and 0.8 (big effect) (Cohen, 1992). G*Power 3.1.9.7 was used to calculate the necessary total sample size (power analysis) to observe an average size effect with a Type I error rate of 0.05 α=0.05 and a power of 0.8 (Type II error rate is β=0.2) for the effect of condition. It was expected that the results from different conditions to be somewhat correlated (r = 0.4) and assume that sphericity assumptions are met. Results for the power analysis for the effect of Condition show that a total of 12 participants are required to observe a significant average size interaction.

## Supplementary Method (water displacement)

The following measurements were made of the hand, forearm, and shoulder volumes. Initially, the hand was immersed up to the cylindric volume of the wrist, leaving a mark with Level 1 water. Next, the hand was plunged up to the elbow, leaving a new mark with Level 2, and finally, the hand was immersed in the water-filled cylinder along the shoulder, leaving a new mark with Level 3. Because the volume of the wrist makes up less than 1% of the total body volume, it was left out of the calculation. The estimated overall inaccuracy can include this omission.

# Supplementary Figures and Tables

This section includes all supplementary figures and tables. Also, analysis related to statistical power analysis and size effect is provided.

## Supplementary Figures

**
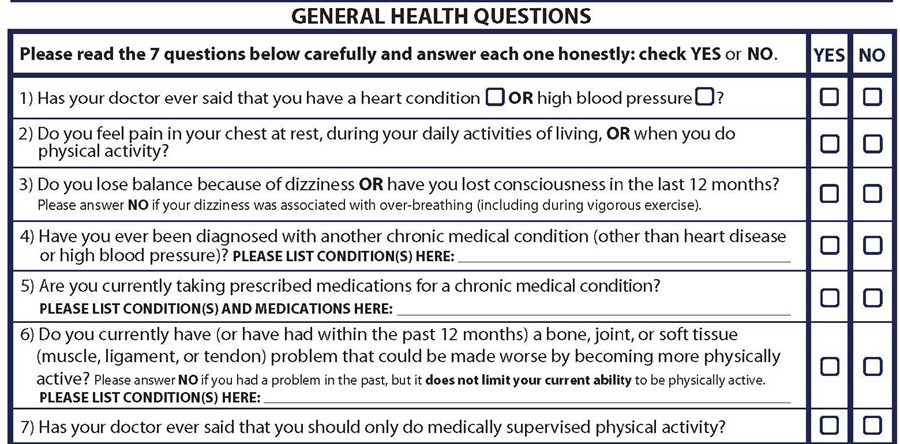
**The questions listed in Supplementary Figure 1 were asked before each experiment.

Supplementary Figure 1. The 2020 Physical Activity Readiness Questionnaire for Everyone (PAR-Q+) (Warburton et al., 2019).

The body segments of participants, such as the whole trunk, upper arm, and forearm were measured by the body Plagenhoef's segmentation model, Supplementary Figure 2.


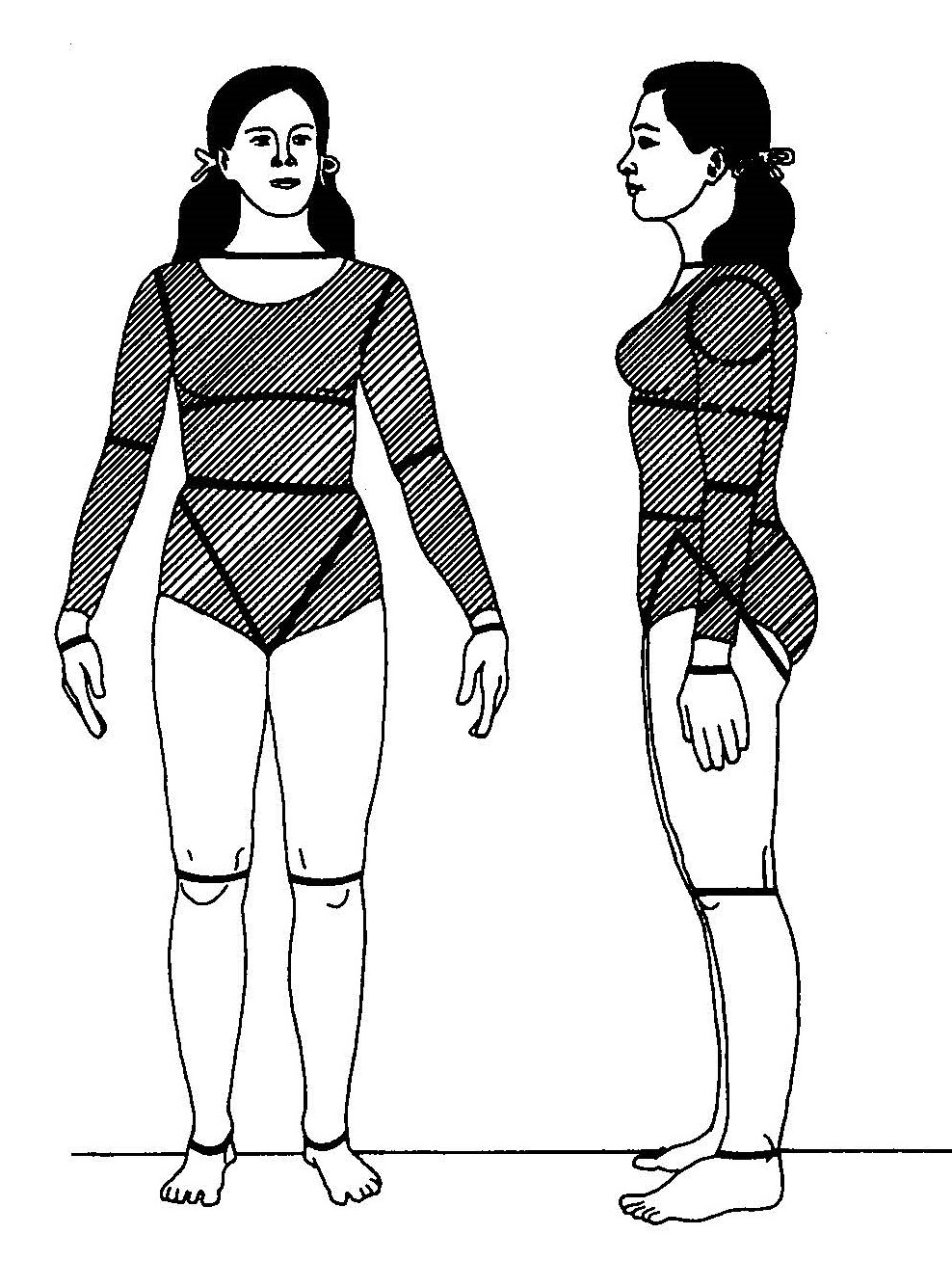


Supplementary Figure 2 Sixteen body segments (Plagenhoef & Abdelnour, 1983). Anatomical Data for Analyzing Human motion, Research Quarterly for Exercise and Sport, Stanley Plagenhoef, F. Gaynor Evans & Thomas Abdelnour (1983) copyright © the Society of Health and Physical Educators, reprinted by permission of Informa UK Limited, trading as Taylor & Francis Group, www.tandfonline.com on behalf of Society of Health and Physical Educators.

## Supplementary Tables

The segment’s masses were calculated concerning the statistical data presented in Supplementary Table 1.

Supplementary Table 1 Segment masses as percentages of total body mass for males and females (Plagenhoef & Abdelnour, 1983). Anatomical Data for Analyzing Human motion, Research Quarterly for Exercise and Sport, Stanley Plagenhoef, F. Gaynor Evans & Thomas Abdelnour (1983) copyright © the Society of Health and Physical Educators. Reprinted by permission of Informa UK Limited, trading as Taylor & Francis Group, www.tandfonline.com on behalf of www.shapeamerica.org.

| One segment | Men N = 35 | | Women N = 100 | |
| --- | --- | --- | --- | --- |
|  | Mean | SD | Mean | SD |
| Hand | 0.65 | 0.06 | 0.5 | 0.026 |
| Forearm | 1.87 | 0.2 | 1.57 | 0.1 |
| Upper arm | 3.25 | 0.49 | 2.9 | 0.32 |
| Foot | 1.43 | 0.13 | 1.33 | 0.02 |
| Shank | 4.75 | 0.53 | 5.35 | 0.47 |
| Thigh | 10.5 | 1.21 | 11.75 | 1.86 |
| Whole trunk | 55.1 | 2.75 | 53.2 | 4.64 |
| Head and neck | 8.26 |  | 8.2 |  |
| Thorax | 20.1 |  | 17.02 |  |
| Abdomen | 13.06 |  | 12.24 |  |
| Pelvis | 13.66 |  | 15.96 |  |

According to Plagenhoef & Abdelnour (1983), for males, the center of mass (CoM) for the forearm, upper arm, and the whole trunk is equivalent to 43%, 43.6%, and 63%, respectively, and for females, it is 43.4%, 45.8%, and 56.9% from the proximal end. The length of the whole trunk is 100% from the hip joint to the shoulder.

Supplementary Table 2. Shapiro-Wilk normality test results for females/males for static and dynamic tasks with elements of repetition. W-test statistic

| Task | W | p-value |
| --- | --- | --- |
| Spine/vertical -1g | 0.86/0.74 | 0.01/0.01 |
| Spine/vertical - 1/6g | 0.92/0.95 | 0.05/0.14 |
| Upper arm/vertical -1g | 0.96/0.94 | 0.36/0.01 |
| Upper arm/vertical - 1/6g | 0.96/0.92 | 0.38/0.01 |

Supplementary Table 3. The mean angle between the spine/upper arm and vertical for static and dynamic tasks together [deg.]. Gravity level (G-level) means gravity change from 1⁄6g to 1g. Note: ***p<0.1; **p<0.05; *p<0.01.

| Dependent variable | Mean spine/vertical angle | | Mean upper arm/vertical | |
| --- | --- | --- | --- | --- |
|  | Males | Females | Males | Females |
| G-level | -13.45*** (3.61) | -17.81***(2.87) | 24.43***(2.53) | 7.34**(3.36) |
| Task | -2.94 (3.55) | 2.25(2.88) | 3.52(2.50) | 0.26(3.37) |
| Constant | 33.86*** (3.29) | 24.96***(2.57) | 49.84***(2.27) | 60.15***(3.00) |
| Observations | 132 | 66 | 138 | 66 |
| R^2^ | 0.1 | 0.38 | 0.41 | 0.07 |
| Adjusted R^2^ | 0.09 | 0.36 | 0.41 | 0.04 |
| Residual Std. Error | 20.38 | 11.67 | 14.71 | 13.64 |
| F Statistic | 7.29*** | 19.53*** | 47.73*** | 2.39* |

**Note:** Constant – the correlation that cannot be explained by the explanatory variables: g-level and task. This is common for all the observations.

The raw data supporting the conclusions of this article is available in the FigShare repository: <https://figshare.com/s/70904b9571323c11b8fa>
